# Abandonment of pearl millet cropping and homogenization of its diversity over a 40 year period in Senegal

**DOI:** 10.1371/journal.pone.0239123

**Published:** 2020-09-14

**Authors:** Katina F. Olodo, Adeline Barnaud, Ndjido A. Kane, Cédric Mariac, Adama Faye, Marie Couderc, Leïla Zekraouï, Anaïs Dequincey, Diégane Diouf, Yves Vigouroux, Cécile Berthouly-Salazar

**Affiliations:** 1 DIADE, Univ Montpellier, Institut de Recherche pour le Développement, Montpellier, France; 2 Centre d’Etude Régional pour l’Amélioration de l’Adaptation à la Sécheresse (CERAAS), Institut Sénégalais de Recherche Agricole (ISRA), Thiès, Senegal; 3 Laboratoire National de Recherche sur les Productions Végétales (LNRPV), Institut Sénégalais de Recherche Agricole (ISRA), Dakar, Senegal; 4 Laboratoire Mixte International Adaptation des Plantes et microorganismes associés aux Stress Environnementaux (LMI LAPSE), Dakar, Senegal; 5 Université Cheikh Anta Diop (UCAD), Dakar, Senegal; 6 Laboratoire Commun de Microbiologie (LCM), Dakar, Senegal; 7 Unité de Formation et de Recherche Environnement, Biodiversité et Développement Durable, Université du Sine Saloum El Hadj Ibrahima Niass (USSEIN), Kaolack, Senegal; CIRAD, FRANCE

## Abstract

Cultivated diversity is considered an insurance against major climatic variability. However, since the 1980s, several studies have shown that climate variability and agricultural changes may already have locally eroded crop genetic diversity. We studied pearl millet diversity in Senegal through a comparison of pearl millet landraces collected 40 years apart. We found that more than 20% of villages visited in 1976 had stopped growing pearl millet. Despite this, its overall genetic diversity has been maintained but differentiation between early- and late-flowering accessions has been reduced. We also found stronger crop-to-wild gene flow than wild-to-crop gene flow and that wild-to-crop gene flow was weaker in 2016 than in 1976. In conclusion, our results highlight genetic homogenization in Senegal. This homogenization within cultivated pearl millet and between wild and cultivated forms is a key factor in genetic erosion and it is often overlooked. Improved assessment and conservation strategies are needed to promote and conserve both wild and cultivated pearl millet diversity.

## Introduction

Since the 20^th^ century, major agricultural transformations have been taking place alongside social, technical and climatic changes. Agriculture has been striving to keep step with these changes and population growth via specialization in a few major crop species and a handful of varieties [1, 2]. Meanwhile, the first impacts of climate change were felt in the 1980s when the Green Revolution took hold in developing countries [2]. In West Africa, climate change had already led to a 10–20% and 5–15% decrease in pearl millet and sorghum yields, respectively [3].

Different adaptation strategies have been implemented to cope with these socio-agro-climatic changes, including mechanical (fertilizer application, change of practices) and biological (choice of crops and/or varieties) technologies. For instance, a replacement of traditional rice varieties by modern varieties, i.e. from 55% in 1996 to 75% in 1998 in the Philippines was prompted by climatic events [4]. In West Africa, short-cycle crop varieties are now widely grown, sometimes through the adoption of modern cultivars [5–7]. Concerns have been raised about how these agricultural changes over the last century could weaken crop diversity through the loss of crops, varieties or alleles [8, 9].

A meta-analysis, based on 48 studies on 9 field crops (mostly cereals) and mostly from North America and Europe but also from South America, Asia and Australia, suggested that genetic diversity has not been markedly impacted in terms of allelic richness [10], but instead quantitative changes occurred with the replacement of pre-existing alleles by new ones [5, 7, 11–14]. Two key aspects have not been investigated: 1) how genetic diversity structure has evolved, and 2) how wild forms have shaped cultivated diversity. Most studies have investigated how varieties have genetically changed over time [5, 15] while not focusing on their genetic structure. We seek to investigate if genetic differentiation between landraces is lower now than they were decade’s ago. Genetic differentiation could reflect specialization to local/marginal conditions. For instance, a multitude of sorghum varieties have been grown in Mali in response to the heterogeneous soil conditions [16]. Mixtures of early- and late-flowering varieties are also an adaptive response to the climatic uncertainty during the growing season [17, 18]. Genetic homogenization would therefore reflect a loss of adaptive potential.

The second aspect that has yet to be properly addressed is how wild relatives—when still locally present—have impacted cultivated diversity. Wild relatives often grow in harsher conditions than cultivated crops [19–22] and are considered as a reservoir of adaptations. Through the introgression of wild adaptations, wild-to-crop gene flow could be an important climate change mitigation strategy/process [23, 24]. By their practices, farmers may—consciously or not—promote gene flow with wild relatives, thereby limiting the loss of diversity while increasing their potential to adapt to on-going changes.

Pearl millet (*Cenchrus americanus* (L.) Morrone syn. *Pennisetum glaucum* (L.) R. Br.) is a major staple cereal in the Sahel. This region has been undergoing severe droughts since the 1990s, notably in Senegal [25–27] with a return of rainfall since 2010 [28] but associated with high local variability [26]. Along with these changes, in Senegal, strong national policies have been implemented since the 1980s to foster food self-sufficiency by inducing farmers to decrease cash-crop production (principally groundnuts) and increase food-crop production (millet, sorghum) through the sale of fertilizer and improved cereal varieties on credit [29–31]. In addition, national improvement programmes were developed to shorten crop cycles [32]. From the 1960s onwards, recurrent selection of local landraces gave rise to the Souna 3 variety with a 90 to 95-day cycle, which led to its gradual adoption throughout the south-central part of the country in the 1970s [33, 34]. Improvement programmes subsequently focused on enhancement of the grain-to-straw ratio and very short crop cycles. Some improved varieties such as IBV-8004, with a 75-day cycle, were tested and released in northern Senegal from 1980 to 1985. There are currently seven registered varieties in the official national catalogue, the entirety is considered early flowering varieties. Nevertheless, despite the extension campaigns, it is assumed that the improved variety adoption rate is still very low [35, 36]. In the groundnut growing area, the region with the highest millet production, the improved variety adoption rate was estimated at 13%, but 27% of farmers had tested and subsequently abandoned them, and most probably creolising them [36].

The aim of this study is to investigate the evolution of the genetic diversity and structure of pearl millet by comparing the diversity found in Senegalese accessions collected in 1976 to those collected in 2016, thus including the pivotal period of major climatic and socio-agricultural changes. Moreover, as Senegal is viewed as a pearl millet diversity hotspot, partly due to the high extent of gene flow with local wild populations [37], we also investigated how wild-crop gene flow evolved over this 40-year period.

## Materials and methods

### Plant materials and sampling strategy

This study was conducted jointly with the Senegalese Institute of Agricultural Research (ISRA), which has a national mandate to collect and conserve all plant genetic resources for food and agriculture in Senegal. During field surveys in 2016, regional and departmental services were informed and, subsequently, they participated in some of the collections. Once we arrived in villages, we first explained the project objectives to local authorities, who then authorized the survey in the village. We then obtained the “prior and informed consent” of each farmer participating in this study, in accordance with the Code of Ethics of the International Society of Ethnobiology.

**Fig 1:**
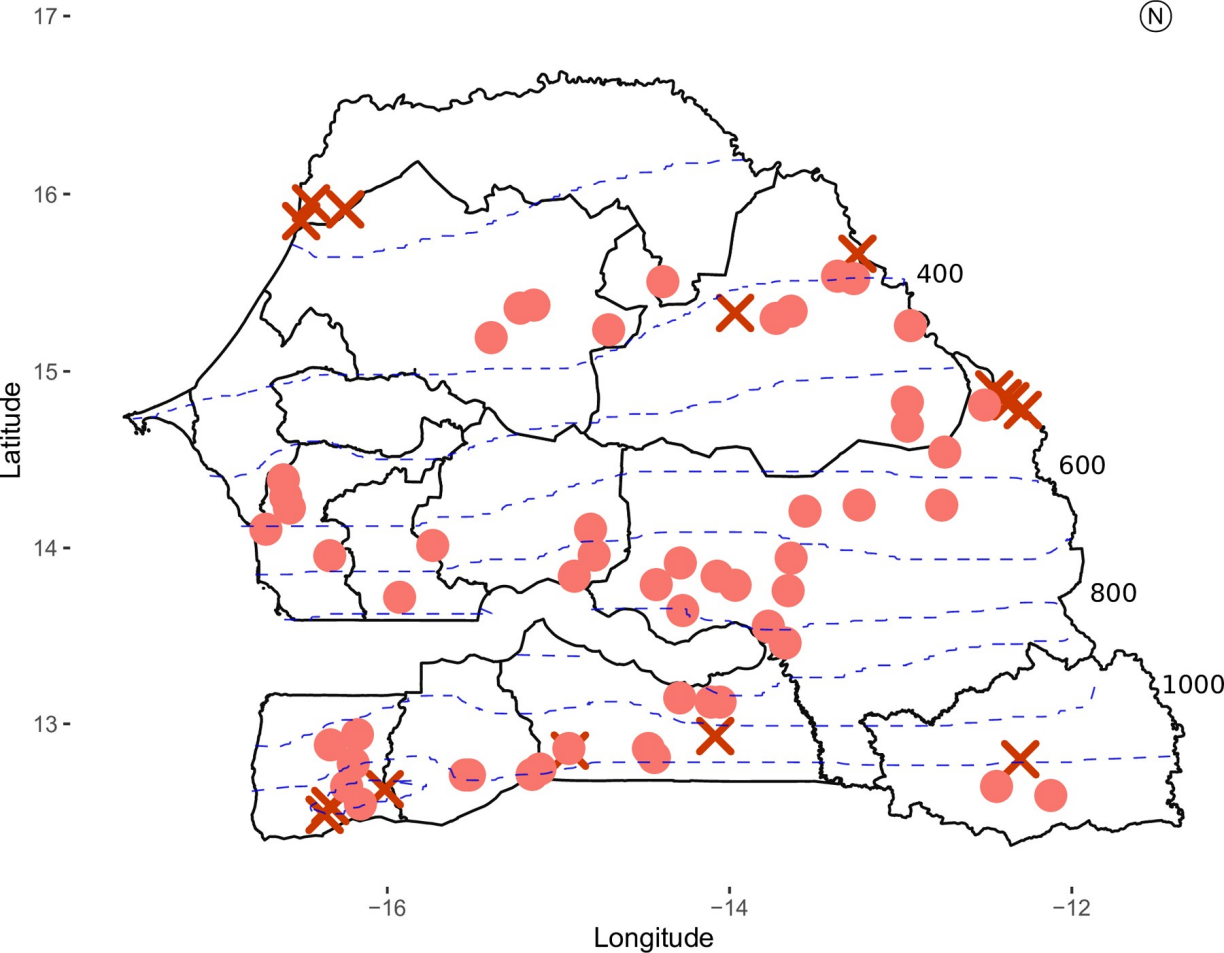
Geographical distribution of cultivated and wild pearl millet accessions used in the analysis. Circles depict surveyed villages that are still cultivating pearl millet; crosses depict surveyed villages with no pearl millet cultivation in 2016. Blue dashed lines represent the isohyets averaged for the 1991–2010 according to [39].

Cultivated pearl millet sampling was first conducted in 1974–1976 and then in 2016 in Senegal. The 1976 collection [38] was conserved at National Research Institute for Sustainable Development (IRD) in France. For the 1976 collection, villages’ geographical coordinates had been manually estimated on maps by first collection agents according to villages’ names. We conducted new surveys from February to April 2016 and visited 69 sites where samples had been collected in 1976 (Fig 1). We were only able to survey 50 sites with the initial coordinates because some villages had moved and/or disappeared. For each of the remaining 19 sites, we visited a village within a 10 km radius of the initially surveyed village.

We first interviewed farmers to ensure that they had not changed their landraces since 1974–1976. This meant that farmers had always self-produced their seed without any voluntary introduction of seed from outside (purchase, donation). We found that farmers only fulfilled this condition in 43 villages out of 69 total sites. We sampled a maximum of five farmers from these villages, identical to the 1974–1976 sampling campaign. Two spikes or a handful of seeds were sampled from each farmer’s granaries, and these samples were subsequently combined to constitute the final village pooled sample. Out of these 43 villages surveyed in 2016, 13 of their respective samples collected in 1974–1976 had good germination rates (> 80%) (S1 Fig). Among these 13 villages, 11 were growing early-flowering (EF) landraces (i.e Souna type) and two villages were growing late-flowering (LF) landraces (i.e Sanio type).

In addition to this cultivated pearl millet collection over a 40-year timespan, wild pearl millet populations (*Pennisetum glaucum monodii* (L.) R. Br.) were sampled at different sites across Senegal from September to October 2015 (S1 Fig). Wild pearl millet can be easily distinguished from cultivated pearl millet by their smaller spike (~10 cm) and small plant size with numerous tillers. A total of 35 wild accessions were sampled based on their wild phenotype and distance from cultivated fields. No wild populations were found at longitudes above -14.91 (S1 Fig).

### DNA extraction and microsatellite amplification

DNA extraction was carried out according to an MTAB protocol, as previously described in [40], from wild pearl millet inflorescences and cultivated pearl millet leaves after plants were grown from seeds in a greenhouse. Twelve polymorphic SSRs were used for genotyping, as described in [41]. A total of 271 individuals from the 1976 pearl millet collection (i.e. 21 individuals/accession on average; range 12 to 29), 309 individuals from the 2016 pearl millet collection (i.e. 24 individuals/accession on average; range 6 to 31), and 642 individuals from the wild pearl millet collection (i.e. 18 individuals/accession on average; range 3 to 36) were kept for subsequent analysis. The average percentage of missing data per individual was 1.75% (S1 Table).

### Diversity statistics estimation

We used FSTAT v.2.9.3.2 [42] to calculate the observed (H_Obs_) and unbiased expected (H_Exp_) heterozygosity, rarefied allelic richness (A_R_), inbreeding coefficient (F_IS_) and differentiation index (F_ST_). We also tested for significant differences in the genetic diversity parameters (H_Exp_, H_Obs,_ A_R,_ F_IS_): i) between wild and cultivated forms, and ii) over sampling years. F_ST_ significance was assessed after 10000 permutations using FSTAT v.2.9.3.2 [42]. Finally, Pearson coefficient correlations were used to assess relationships between geographic coordinates, ancestry and genetic diversity parameters.

### Genetic structure

A panel of complementary methods were used to assess genetic structure within and between cultivated and wild forms. We performed scaled and centred principal component analyses (PCA) using the FactomineR package v.1.4.2 [43] and factoextra package v.1.0.5 [44] in the R environment [45] v.3.6.0. Then, we conducted Bayesian clustering analyses in STRUCTURE v.2.3.4 [46] using the admixture model [47] and correlated allele frequency option, with a burn-in period of 100000 steps and 500000 MCMC replicates. Ten independent runs were performed for each K, ranging from K = 1 to K = 10. The most probable K value was determined by the *D*.*ΔK* criterion [48] and the log-likelihood (Ln P (D | K)) plot with STRUCTURE HARVESTER v0.6.94 [49]. To investigate further the wild pearl millet genetic structure, we incorporated spatial information by performing a spatial principal component analysis (sPCA) implemented in the adegenet package v.2.1.1 [50] in the R environment [45] v.3.6.0. We used a Gabriel graph as spatial connection network.

## Results

### Pearl millet growing area

In our survey, we found that 78% (54/69) of the initially surveyed villages were still growing pearl millet in 2016, out of which 16% (11/69) had introduced new varieties. Farmers from over 20% of the villages visited in 1976 had stopped growing pearl millet. Those villages were located above 14°N and below 13°N (Fig 1). Most reasons put forward by the farmers to explain this choice were: insufficient rainfall, pest and bird attacks, and poor soil fertility (pers. observations).

### Wild and cultivated pearl millet differentiation

**Fig 2:**
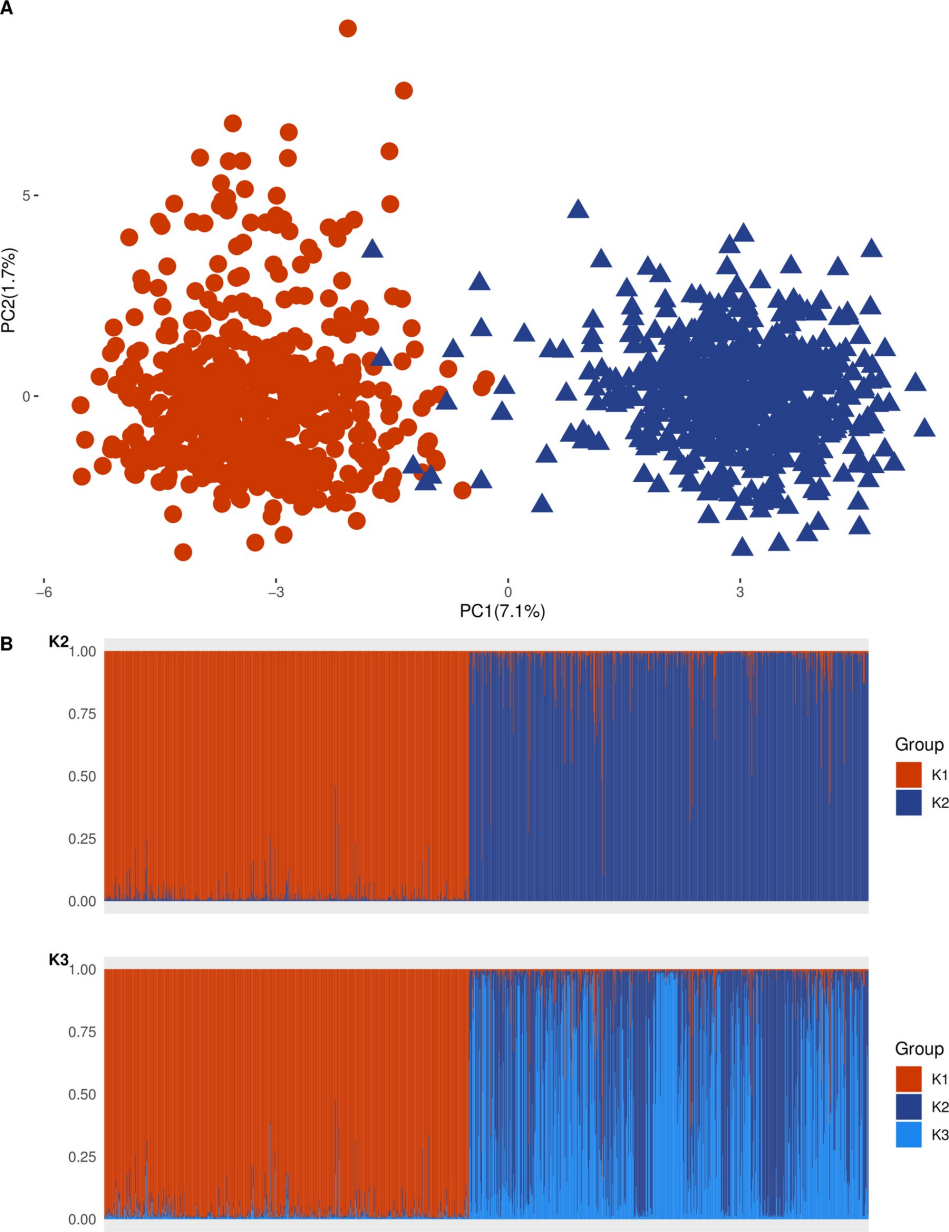
Genetic structure of cultivated and wild pearl millet accessions. (A) Principal component analysis results. (B) Inference of populations determined by STRUCTURE. Cultivated pearl millet including 1976 and 2016 collections is presented in red and wild pearl millet is presented in blue. The Structure results are shown for K = 2 (see S2 Fig for the most likely cluster) and K = 3. Each sample is represented by a vertical line partitioned within the K colored group corresponding to the proportion of genome assigned to each cluster.

Marked differentiation was observed between wild and cultivated pearl millet samples. The first two PCA axes explained 7.1% and 1.7% of the inertia, respectively (Fig 2A). Cultivated and wild accessions were clearly separated on the first axis, while the second reflected genetic differences within cultivated pearl millet. A handful of wild accessions with intermediate positions on the first axis suggested potential hybrids samples. Those accessions were spatially distributed throughout Senegal. The structure analysis findings fit those of the PCA analysis, with the most likely number of clusters being K = 2 (S3 Fig), representing cultivated and wild pearl millet forms (Fig 2B). Ten wild samples fell within the cultivated cluster, with q-values > 0.5. Those individuals were removed from subsequent analysis. Had the individuals not been removed, the study’s main results would remain unaltered. The genetic differentiation F_ST_ between wild and cultivated pearl millet was 0.227 (permutation test, P = 0.001). The cultivated and wild pearl millet samples were not significantly different with reference to the expected heterozygosity (Table 1, Wilcoxon paired test, V = 31, P > 0.05), but they were significantly different for allelic richness A_R_ (Table 1, Wilcoxon paired test, V = 1, P = 0.003). The average population crop-to-wild ancestry (q_cw_) was significantly higher than the wild-to-crop ancestry (q_cw_ = 0.033 *vs*. q_wc_ = 0.019, Mann-Whitney-Wilcoxon test, W = 315, P = 0.041).

**Table 1:** Summary of genetic diversity parameters for cultivated and wild pearl millet.

	N_Ind_	H_Exp_	H_Obs_	A_R_	F_IS_	q_cw_	q_wc_
**Cultivated**	580	0.583 ± 0.157	0.478 ± 0.191	8.2 ± 3.7	0.187 ± 0.196	-	0.019 ± 0.011
**Wild**	642	0.637 ± 0.208	0.516 ± 0.201	11.4 ± 5.7	0.198 ± 0.186	0.033 ± 0.024	-
**P- value**	-	> 0.05 ^a^	> 0.05 ^a^	0.003 ^a^	> 0.05 ^a^	0.041 ^b^	

N_Ind:_ number of individuals; H_Exp_: expected heterozygosity, H_Obs_: observed heterozygosity, A_R:_ allelic richness were rarefied on a minimal sample size of 518 individuals; F_IS_: inbreeding coefficient; q_cw_: mean crop-to-wild ancestry obtained from the STRUCTURE results at K = 2; q_wc_: mean wild-to-crop ancestry obtained from the STRUCTURE results at K = 2. ^a^ P-value calculated by a Wilcoxon paired test and ^b^ P-value calculated by a Mann-Whitney-Wilcoxon test.

### Wild diversity and structure

**Fig 3:**
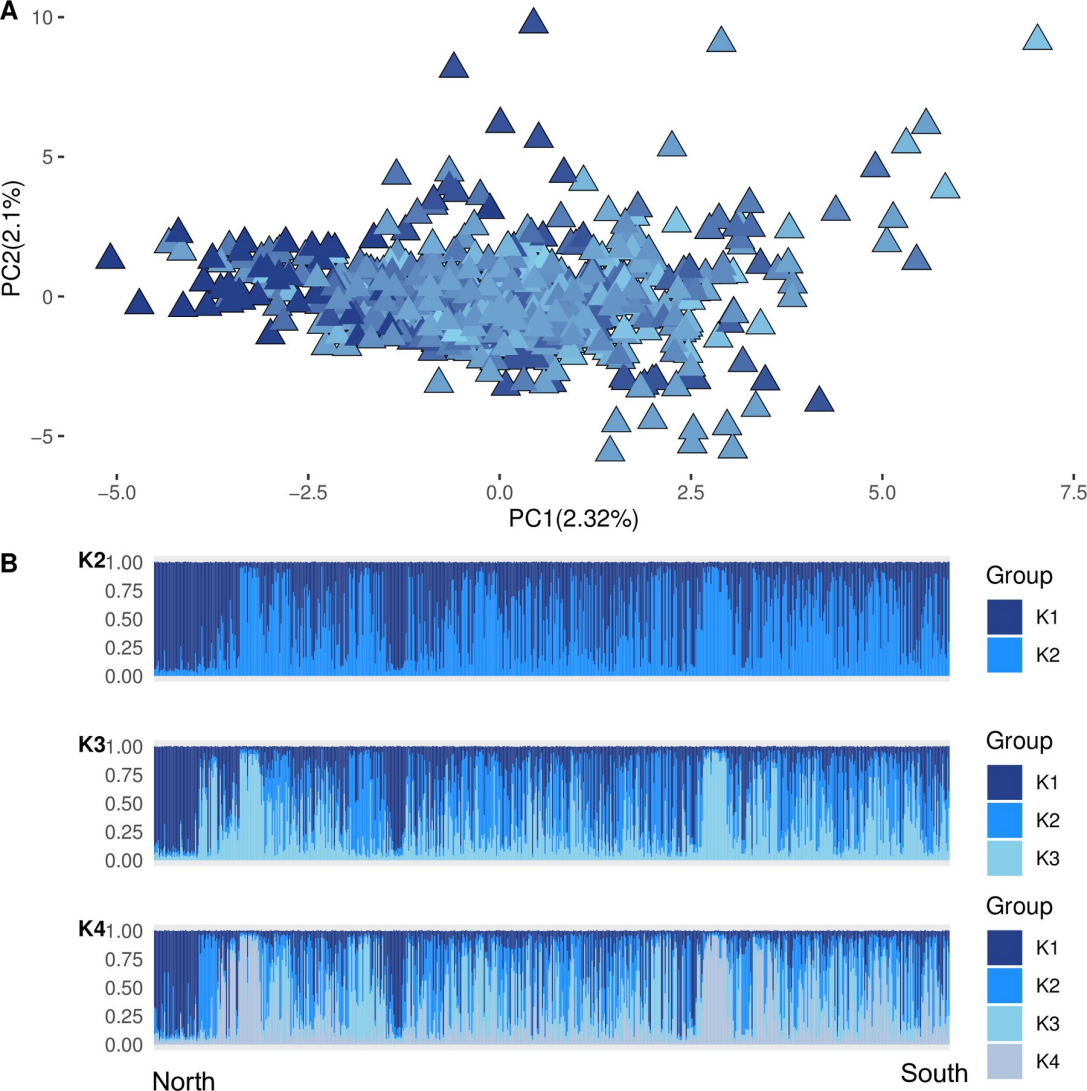
Genetic structure of wild pearl millet. (A) Principal component analysis results. A color gradient from dark blue to light blue indicates a latitudinal north-south gradient. (B) STRUCTURE results for K = 2 (the most likely cluster, see S3 Fig), K = 3 and K = 4. Populations are ordered on a latitudinal north-south gradient. Each individual is represented by a vertical line partitioned within the K colored group corresponding to the proportion of genome assigned to each cluster.

Wild pearl millet accessions showed a weak structure and only slight geographical structuring. We found that PC1 and PC2 summarized 2.3% and 2.1% of the inertia, respectively (Fig 3A). A significant correlation was nevertheless obtained between PC1 and latitude (S2 Table), (r = -0.26, P = 2.27 10^−11^) or longitude (r = -0.24, P = 8.29 10^−10^), thus reflecting a weak spatial effect on wild pearl millet diversity. The STRUCTURE analysis revealed the highest ΔK for K = 2 and K = 4 (S3 Fig). However, no clear groups were identified (Fig 3B).

Adding spatial information as a variable could help in identifying groups when there is very low genetic structuring. However, the spatial PCA did not improve the structuration analysis (S4 Fig). The expected heterozygosity (H_Exp_) averaged 0.607 per site (range 0.463 to 0.669) and the mean allelic richness (A_R_) was 2.3 (range 1.9 to 2.5; Table 2). We found that crop-to-wild ancestry (q_cw_) was negatively correlated with latitude (r = -0.41; P = 0.015) and positively correlated with allelic richness (r = 0.34, P = 0.044) and the expected heterozygosity (r = 0.40; P = 0.018). We noted a nearly significant negative correlation between wild expected heterozygosity and longitude (r = -0.32, P = 0.057; S3 Table and S5 Fig).

**Table 2:** Summary of genetic diversity statistics for wild pearl millet.

Sites	Latitude	Longitude	N_Ind_	H_Exp_	H_Obs_	A_R_	F_IS_	q_cw_
**Niango_Dieu**	**16.511**	**-14.920**	**36**	**0.463**	**0.404**	2.0	0.087	0.007
**Ndiol**	16.167	-16.287	33	0.575	0.487	2.2	0.062	**0.005**
**Mala_Tak**	16.135	-15.881	15	0.523	0.417	2.1	0.206	0.011
**Mpal**	15.919	-16.287	25	0.636	0.571	2.4	0.077	0.039
**Louga**	15.708	-16.069	33	0.656	0.601	**2.5**	0.028	0.035
**Niomre**	15.666	-16.098	15	0.564	0.554	2.2	0.128	0.007
**Gande**	15.534	-15.929	29	0.638	0.548	2.4	0.030	0.077
**Loyene**	15.483	-15.760	16	0.568	0.558	2.2	0.119	0.012
**Ndoyene**	15.434	-15.760	14	0.668	0.614	**2.5**	0.197	0.027
**Kebemer**	15.419	-16.404	16	0.653	0.514	**2.5**	0.145	0.007
**Ndiagne**	15.387	-16.074	11	0.567	0.536	2.2	0.108	0.018
**Afe**	15.214	-15.560	14	0.620	**0.624**	2.4	**0.005**	0.032
**Sagata_Djolof**	15.213	-15.563	21	0.598	0.550	2.3	0.125	0.069
**Pekes**	15.112	-16.425	20	0.633	0.588	2.4	0.018	0.028
**Darou_Mousti**	15.099	-16.058	17	0.585	0.536	2.3	0.167	0.012
**Mekhe**	15.093	-16.657	13	0.621	0.575	2.3	0.206	0.037
**Thilmaka**	15.040	-16.248	17	**0.669**	0.585	**2.5**	0.107	0.019
**Daobe_Edil**	15.036	-15.785	16	0.634	0.530	2.4	0.150	0.053
**Touba_Belel**	15.007	-15.873	16	0.627	0.594	2.4	0.043	0.035
**Keur_Samba_Kane**	14.886	-16.596	18	0.633	0.573	2.4	**0.218**	0.015
**Banghadj**	14.863	-16.658	27	0.635	0.554	2.4	0.128	0.019
**Lac_rose**	14.838	-17.241	21	0.622	0.551	2.4	0.087	0.018
**Mboubane_Ndiaye**	14.832	-16.571	19	0.528	0.500	2.1	0.102	0.020
**Touba**	14.827	-15.936	21	0.647	0.622	2.4	0.133	0.030
**Thiès**	14.770	-16.847	23	0.601	0.541	2.3	0.089	0.040
**Dakar**	14.729	**-17.206**	15	0.659	0.572	**2.5**	0.056	0.052
**Diourbel**	14.657	-16.232	13	0.584	0.529	2.3	0.106	0.006
**Poultock**	14.528	-16.502	14	0.611	0.539	2.3	0.082	0.033
**Mbabane_Wolof**	14.486	-15.259	14	0.602	0.480	2.3	0.053	0.048
**Fasse**	14.361	-16.036	17	0.618	0.604	2.4	0.142	0.038
**Fayil**	14.328	-16.493	19	0.626	0.577	2.4	0.089	0.076
**Ndiokène**	14.297	-15.190	9	0.582	0.476	2.2	0.162	0.052
**Delby**	14.250	-15.302	29	0.614	0.556	2.4	0.087	0.037
**Diofor**	14.164	-16.662	**3**	0.556	0.472	**1.9**	0.102	0.013
**Sambadia**	**14.130**	-16.695	**3**	0.632	0.583	2.3	0.077	**0.082**
**Mean ± standard deviation**			18 ± 7	0.607 ± 0.045	0.546 ± 0.052	2.3 ± 0.1	0.106 ± 0.054	0.032 ± 0.021

N_Ind_: number of individuals; H_Exp_: expected heterozygosity; H_Obs_: observed heterozygosity; A_R_: allelic richness was rarefied on a minimal sample size of two individuals.; F_IS_: inbreeding coefficient, q_cw_: mean crop-to-wild ancestry obtained from the STRUCTURE results at K = 2. The range of each column is in bold in the table.

### Temporal evolution of cultivated diversity

We assessed changes in cultivated pearl millet diversity by comparing the findings of the 1976 and 2016 collections. For early-flowering groups (EF), we found 88 alleles in 1976 datasets and 90 alleles in 2006 datasets; similarly, for late-flowering groups (LF), we found 52 alleles and 47 alleles, respectively. For EF, seven private alleles were lost while nine were gained, and for LF more private alleles were lost than gained (11 *vs*. 6, S6 Fig). In terms of genetic diversity parameters, we found a slightly significant decrease in allelic richness between EF-76 and EF-16 (A_R___EF-76_ = 5.1 *vs*. A_R___EF-16_ = 4.9, Wilcoxon paired test, V = 56, P = 0.045). For LF accessions, a significant decrease was found only for H_Obs_ (H_Obs_LF-76_ = 0.464 *vs*. H_Obs_LF-16_ = 0.369, Wilcoxon paired test, V = 78, P = 0.002, Table 3).

**Table 3:** Summary of genetic diversity statistics for the early- and late-flowering accessions according to the sampling year.

	N_Ind_	H_Exp_	H_Obs_	A_R_	F_IS_	q_wc_
**EF-76**	243	0.575	0.481	5.1	0.164	0.019
**EF-16**	290	0.575	0.483	4.9	0.16	0.018
**P- value**	*-*	> 0.05 ^a^	> 0.05 ^a^	0.045 ^a^	> 0.05 ^a^	0.021 ^b^
**LF-76**	28	0.511	0.464	3.9	0.113	0.028
**LF-16**	19	0.521	0.369	4	0.279	0.014
**P- value**	*-*	> 0.05 ^a^	0.002 ^a^	> 0.05 ^a^	> 0.05 ^a^	0.148 ^b^

N_Ind:_ number of individuals; H_Exp_: expected heterozygosity; H_Obs_: observed heterozygosity; A_R:_ allelic richness was rarefied on a minimal sample size of 18 individuals; F_IS_: inbreeding coefficient; q_wc_: mean wild-to-crop ancestry obtained in the STRUCTURE results at K = 2. ^a^ P-value calculated by a Wilcoxon paired test and ^b^ P-value calculated by a Mann-Whitney-Wilcoxon test.

**Fig 4:**
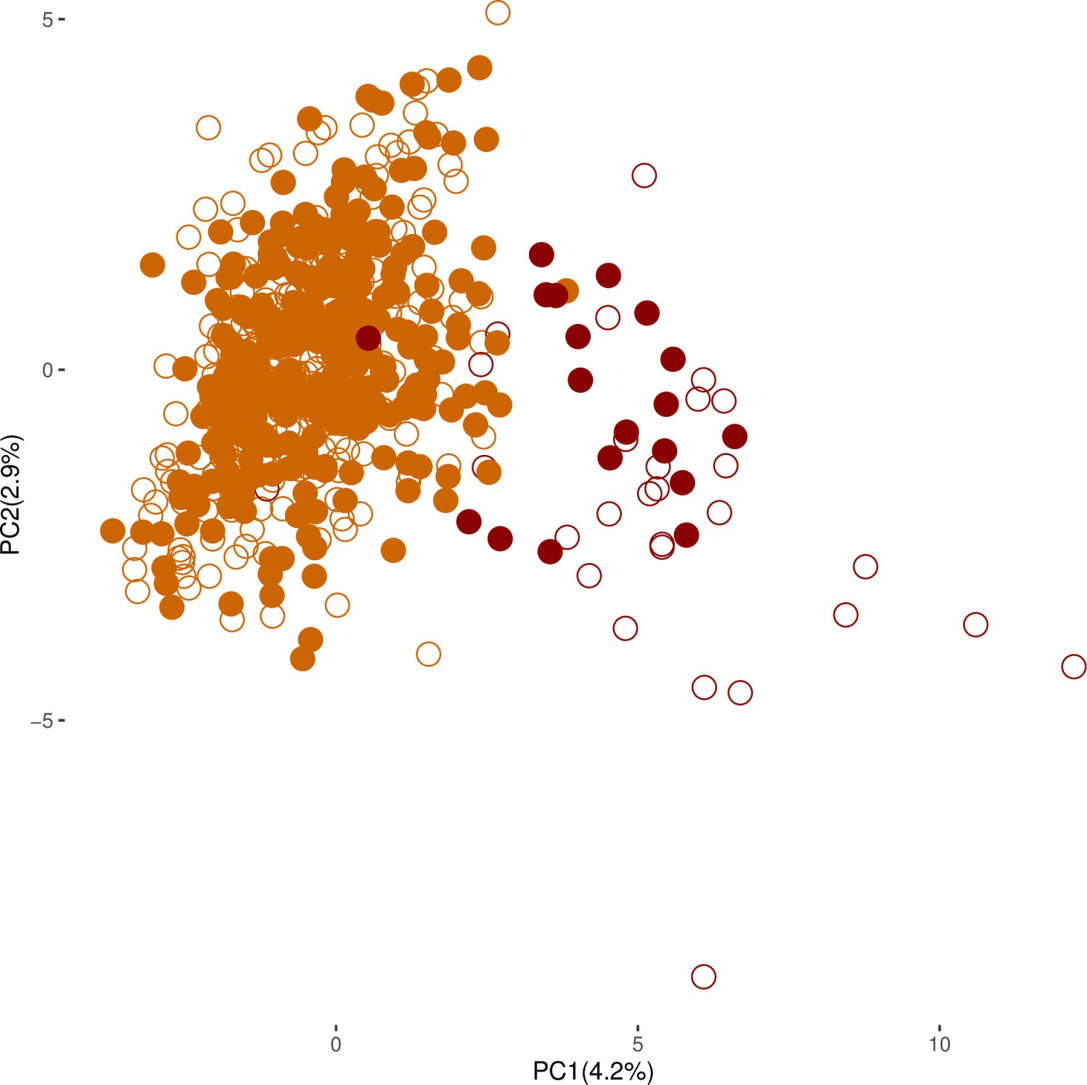
Temporal principal component analysis of cultivated pearl millet. Empty circles indicate the 1976 collection and solid circles indicate the 2016 collection. Early-flowering accessions (EF) are presented in orange and late-flowering (LF) accessions are presented in dark red.

The cultivated pearl millet genetic structure changed over time. The PCA showed that PC1 and PC2 jointly accounted for 7.1% of the total variance (Fig 4). PC1 clearly separated the EF and LF accessions for both collection years. For the 2016 EF and LF accessions, the PC1 values differed significantly from those of 1976 (Mann-Whitney-Wilcoxon test, W = 31570 for EF, W = 361 for LF, P < 0.05) and were close to zero, suggesting genetic convergence between the two groups. PC2 represented the within group diversity. For LF accessions, PC2 variance was significantly reduced by twofold in 2016 compared to 1976 (1.88 *vs*. 4.59, F-test, F = 2.44, P = 0.052), suggesting a higher genetic homogenization in 2016. In addition to these results, the mean pairwise F_ST_ values seemed to decrease in 2016 (F_ST-76_ = 0.069 *vs*. F_ST-16_ = 0.059, but not significantly according to the paired Wilcoxon test, V = 1836.5, P = 0.141). A significant reduction was noted between flowering groups, i.e. the mean pairwise F_ST_ between EF and LF went from 0.180 in 1976 to 0.147 in 2016 (paired Wilcoxon test, V = 197.5, P = 0.022, Table 4).

**Table 4:** Mean pairwise F_ST_ between and within EF and LF accessions.

	Year-1976	Year-2016	P-value
**Mean pairwise EF**	0.024	0.024	-
**Mean pairwise LF**	0.089	0.035	-
**Mean pairwise EF *vs* LF**	0.180	0.145	0.022
**Mean pairwise comparison**	0.069	0.059	0.141

P-values were calculated using a Wilcoxon paired test.

**Fig 5:**
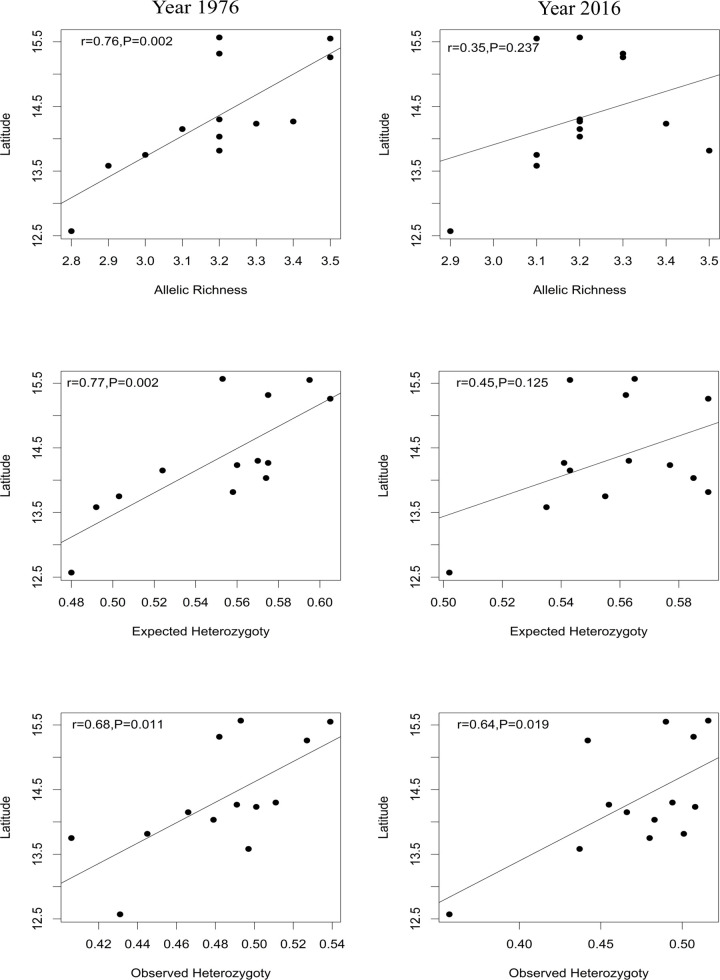
Correlations between latitude and H_Exp_, H_Obs_ and A_R_ according to the sampling year.

Next, we focused on the spatial structure’s evolution over time by correlating the genetic parameters with the geographical coordinates. In 1976, we observed a significant positive correlation between latitude and allelic richness (r = 0.76, P = 0.002), expected heterozygosity (r = 0.77; P = 0.002) and observed heterozygosity (r = 0.68; P = 0.011; Fig 5, S3 Table). These correlations were no longer significant in 2016 for allelic richness (r = 0.35, P = 0.237), expected heterozygosity (r = 0.45, P = 0.125), but were still significant for observed heterozygosity (r = 0.64, P = 0.019).

Lastly, we found that the mean wild ancestry in cultivated forms slightly decreased from 1976 to 2016 (mean q_wc-76_ = 0.021 *vs*. mean q_wc-16_ = 0.018, Mann-Whitney-Wilcoxon test, W = 47118, P = 0.008). A more in-depth analysis revealed significant findings for early flowering accessions (mean q_wc-76_ = 0.019 *vs*. mean q_wc-16_ = 0.018, Mann-Whitney-Wilcoxon test, W = 39450, P = 0.021).

## Discussion

### Abandonment of pearl millet cropping in Senegal

A staggering proportion of villages (20%) were found to have abandoned pearl millet cropping mainly at latitudes above 14°N. Several studies have reported evidence of the adoption and southward displacement of short-cycle cultivars in response to the Sahelian drought of 1980 [26, 27] associated with a 0.9°C temperature increase since 1975 [27] and declining rainfall in West Africa [6, 51–53], yet ours is the only study that highlights the complete abandonment of pearl millet cropping. We noted this pattern in regions at latitudes above 14°N, i.e. in the Saint-Louis, Louga and Matam regions. The need for varieties with very short cycles (< 60–75 days; [54]) had been stressed since the 1970s for these regions. Except for Saint-Louis where the cultivated area has increased due to the introduction of intensive Asian rice cultivation policies, Louga and Matam lost 12% to 18% of their overall cultivated area between 2001 and 2017 [55], including 50% of the pearl millet cropping area.

Similarly, we observed pearl millet cropping abandonment in southern villages under suitable rainfall conditions. The rice, maize, cassava and cowpea cultivated area has increased by more than fourfold in this region [55]. Most of these crops are mainly used as cash crops and sold on markets. Pearl millet yields never exceed 0.9 t/ha while rice yields may reach 2 t/ha and cassava yields are sometimes over 10 t/ha [55]. Pearl millet has hence been replaced by more productive crops, in line with the trend in other West African countries [56].

Overall, this suggests that, in recent years, there has been a shift in agricultural strategy from food to cash crops, with a marked reduction in cultivated areas in northern Senegal. Despite the observed changes in the pearl millet growing area and the magnitude of drought events, we did not find a significant decrease in terms of genetic diversity between the two periods. Two non-exclusive explanations can be put forward: 1) pearl millet is a very diversified allogamous species, and 2) despite some seed loss events, which were probably very localized, diversity has likely been maintained through the reacquisition of landraces via traditional seed exchange systems [57–61].

### Homogenization of pearl millet diversity across Senegal

Neutral diversity has been maintained but its structure has changed. Our results revealed homogenization within and between early- and late-flowering landraces. We acknowledge that our LF sample size was small due to the germination issues discussed in the Materials and Methods section; nonetheless, homogenization within and between EF and LF landraces was congruent with: 1) the southward displacement of EF landraces, thus increasing the overlap of EF and LF growing areas [53], and 2) the over-representation of EF at the national scale, thus increasing the likelihood of EF to LF gene flow. Genetic homogenization within EF landraces was also revealed by the loss of genetic diversity structuring over a latitudinal gradient. Genetic homogenization could be the result of an intensification of seeds’ exchange between farmers and along larger scales. Another possibility is that homogenization results from the diffusion and release at large scale of improved varieties such as Souna 3 [32, 62]. Improved varieties are not always adopted [36], nevertheless, because farmers test them over a few years, they contribute to local pollen and seed mediated gene flow in local varieties. This could explain the pattern we observed, a large-scale homogenization of diversity.

This genetic homogenization may have major impacts pertaining to agricultural adaptation and resilience. From an agroecological standpoint, there is now a growing body of evidence on the role varietal mixtures could play in adaptation to climate change, notably in terms of resilience, i.e. ensuring minimal production to climate uncertainty [63–65]. While LF landraces are preferred by farmers (higher crop and fodder yields, etc.), these crops are riskier with respect to the uncertainty regarding the rainy season. Farmers may grow EF and LF landraces on the same plot—EF landraces ensure a minimal yield under poor rainy season conditions, whereas LF landraces can make profitable use of late rainfall of the rainy season and produce sufficient fodder [18].

### Wild and cultivated pearl millet gene flow

Weedy pearl millet and sorghum plants can sometimes be used by farmers for their earliness in harsh conditions [66–68], therefore facilitating gene flow between wild and cultivated gene pools. We found that admixture rates were higher in 1976 than in 2016; this phenomenon could possibly be explained by the fact that EF landraces were cultivated further south, which could have reduced the overlap of the wild and cultivated pearl millet distribution area (Fig 1).

Our study also showed that the diversity of wild relatives is at risk. Indeed, cultivated-to-wild pearl millet gene flow was significantly higher, leading to a change in terms of A_R_ and H_Exp_ in the wild diversity, thus increasing the risk of genetic swamping. Similar results have also been obtained in the Sahelian region [69]. When sampling, we sought avoiding weedy phenotypes and removed miss-assigned samples (potential weeds) from analyses. Estimates were therefore conservative, and could be considered as the lower limit of the estimate of contamination of the wild pool by the cultivated pearl millet pool.

Wild relatives are seen as a reservoir for future adaptation [24, 70], their importance in climate change mitigation strategies is increasingly recognized [71]. Until recently, cultivated alleles were thought to be maladaptive and counter-selected very quickly. It has now been demonstrated that cultivated diversity could be beneficial and thus positively selected in wild conditions [72, 73]. Despite their value, crop wild relatives (CWR) are generally poorly represented in *ex situ* collections [74, 75] and efforts ought to be encouraged to conserve more wild diversity in gene banks. In Senegal, the low pairwise F_ST_ noted between wild populations (S5 Table) confirmed the lack of structuring, which means that a limited number of accessions would be enough to conserve wild diversity. *Ex-situ* collection could also enable time-course studies and help gain insights into wild pearl millet evolution [76, 77].

Genetic homogenization might negatively impact long-term adaptation [78]. If improved varieties contribute to the local maintenance or increase of genetic diversity [9], their large dispersal is likely to lead to a strong spatial homogenisation of diversity [78, 79]. Recent methodological developments make it possible to measure the adequacy of the diversity currently available to survive in the future climatic conditions, i.e. the genomic vulnerability [80, 81]. These approaches developed in trees [80] and birds [81] allow identifying diversity crucial for the adaptation to future climate conditions. Conservation strategies ought to focus on enhancing the functional diversity from wild and cultivated populations in current and future gene banks. It is essential to improve quantification of not only diversity but also erosion of the functional diversity. Characterizing erosion of functional diversity will make possible to determine the real impact of the large-scale dissemination of few improved varieties and thus influence the rationale underlying agricultural development policymaking.

## Conclusion

First, this study is one of the only studies documenting a decrease of the pearl millet growing area. With an even drier future climate, pearl millet cultivation might continue to decrease in the northern part of Senegal. A strong homogenisation of diversity is now observed across Senegal, likely hampering abilities of pearl millet to adapt to future climate. Two major strategies for increasing diversity in pearl millet and favouring its adaptation to climate changes ought to be implemented: 1) better define plant breeding/variety development programs and appropriate seed dissemination and 2) development of strategies on assisted migration. Assisted migration, i.e. introducing crops/varieties from areas where current ecological niches could correspond to those of the target region in the future, can be a major adaptive strategy to cope with future climatic conditions. However, this approach may not be suitable in a system where large-scale diversity homogenization is underway, thus highlighting the need to focus on the conservation of functional and local diversity for crops and their wild relatives.

## Supporting information

S1 Fig Geographical distribution of wild and cultivated pearl millet accessions used in the analysis. Blue triangles indicate the 35 wild pearl millet sampling sites. Red circles indicate sampling sites where 13 pairwise collections of cultivated pearl millet were conducted 40 years apart, i.e. 1976 *vs*. 2016.
(TIF)

S2 Fig Graph of delta K values for estimation of the likely number of clusters for the wild and cultivated dataset. The Evanno method implemented in the STRUCTURE HARVESTER program suggested K = 2 as the most likely K value.
(TIF)

S3 Fig Graph of delta K values for estimation of the likely number of clusters for the 35 wild pearl millet populations. The Evanno method implemented in the STRUCTURE HARVESTER program suggested K = 2 as the most likely K value.
(TIF)

S4 Fig Spatial genetic structures inferred by sPCA analysis on the 35 wild pearl millet accessions. (A) Representation of the connexion network (Gabriel graph) used for the sPCA analysis. (B) Plot of the first sPCA axis scores. Scores are depicted over a grey level gradient, with dark grey and white colors for positive and negative scores, respectively.
(TIF)

S5 Fig Correlations between genetic statistics and ancestry, geographical coordinates for wild pearl millet. (TIF)

S6 Fig Change in numbers of alleles within and between flowering landraces in 1976 and 2016. (TIF)

S1 Table Genotypic data for 1223 individuals used in this study. The table header lists the individual identifiers, populations, sampling year, site name, geographical coordinates, form, type of flowering, percentage of missing data, and SSR marker identifiers.
(XLSX)

S2 Table PC1, PC2 coordinates and q_cw_ values of each wild individual. (XLSX)

S3 Table Pearson correlation matrix between geographical coordinates and genetic estimates in wild and cultivated pearl millet according to sampling year. (PDF)

S4 Table Coordinate values for each cultivated individual based on the principal component analysis. (XLSX)

S5 Table Pairwise F_ST_ values in wild populations. F_ST_-values (below diagonal) and P-values (below diagonal).
(XLSX)
