# Supplementary figures and images for: Abandonment of pearl millet cropping and homogenization of its diversity over a 40 year period in Senegal
Source: PLoS One. 2020 Sep 14;15(9):e0239123. doi: 10.1371/journal.pone.0239123 (PMC7489563; doi:10.1371/journal.pone.0239123)

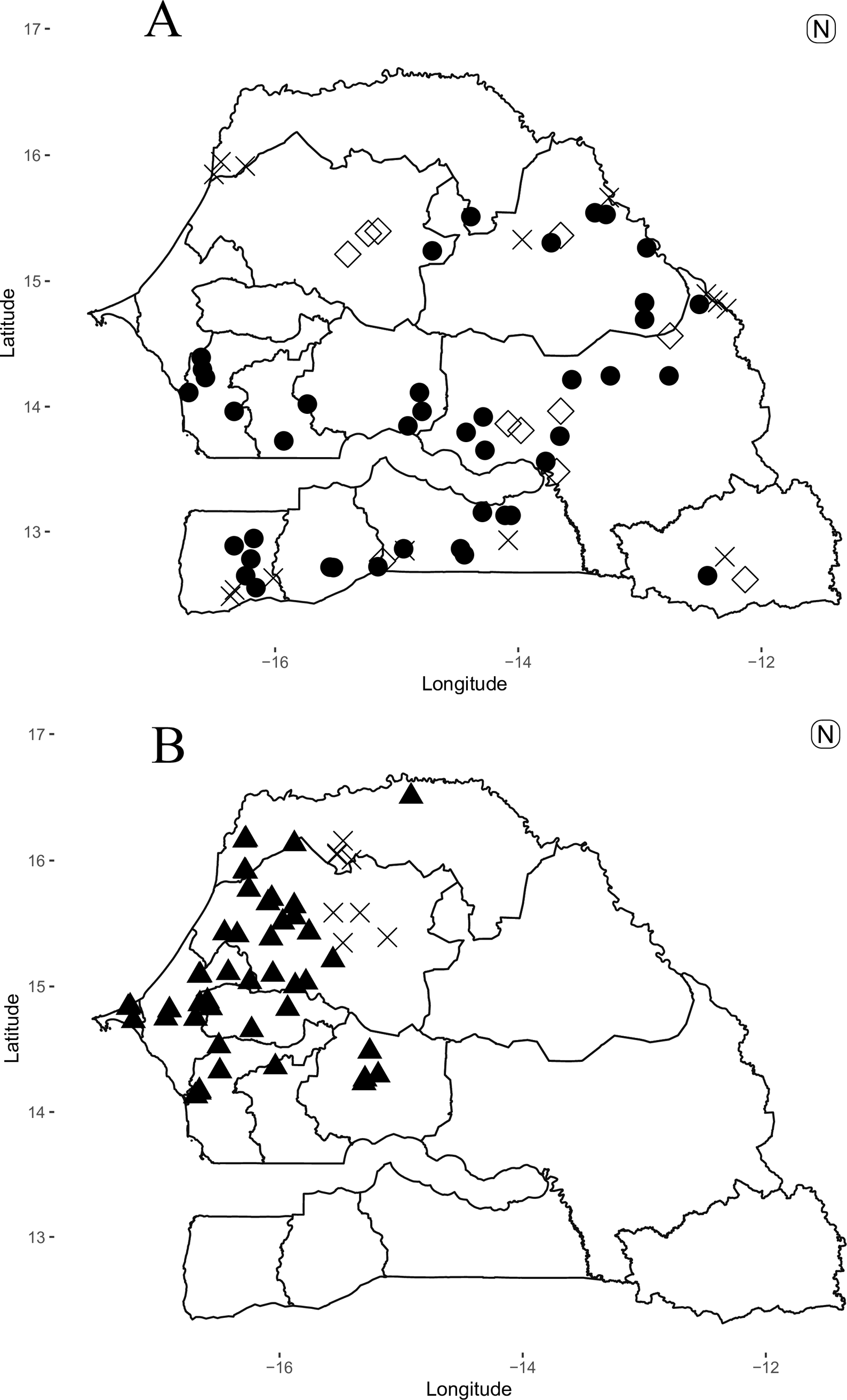

Supplement: S1 Fig — Blue triangles indicate the 35 wild pearl millet sampling sites. Red circles indicate sampling sites where 13 pairwise collections of cultivated pearl millet were conducted 40 years apart, i.e. 1976 vs. 2016. (TIF) [file pone.0239123.s001.tif]

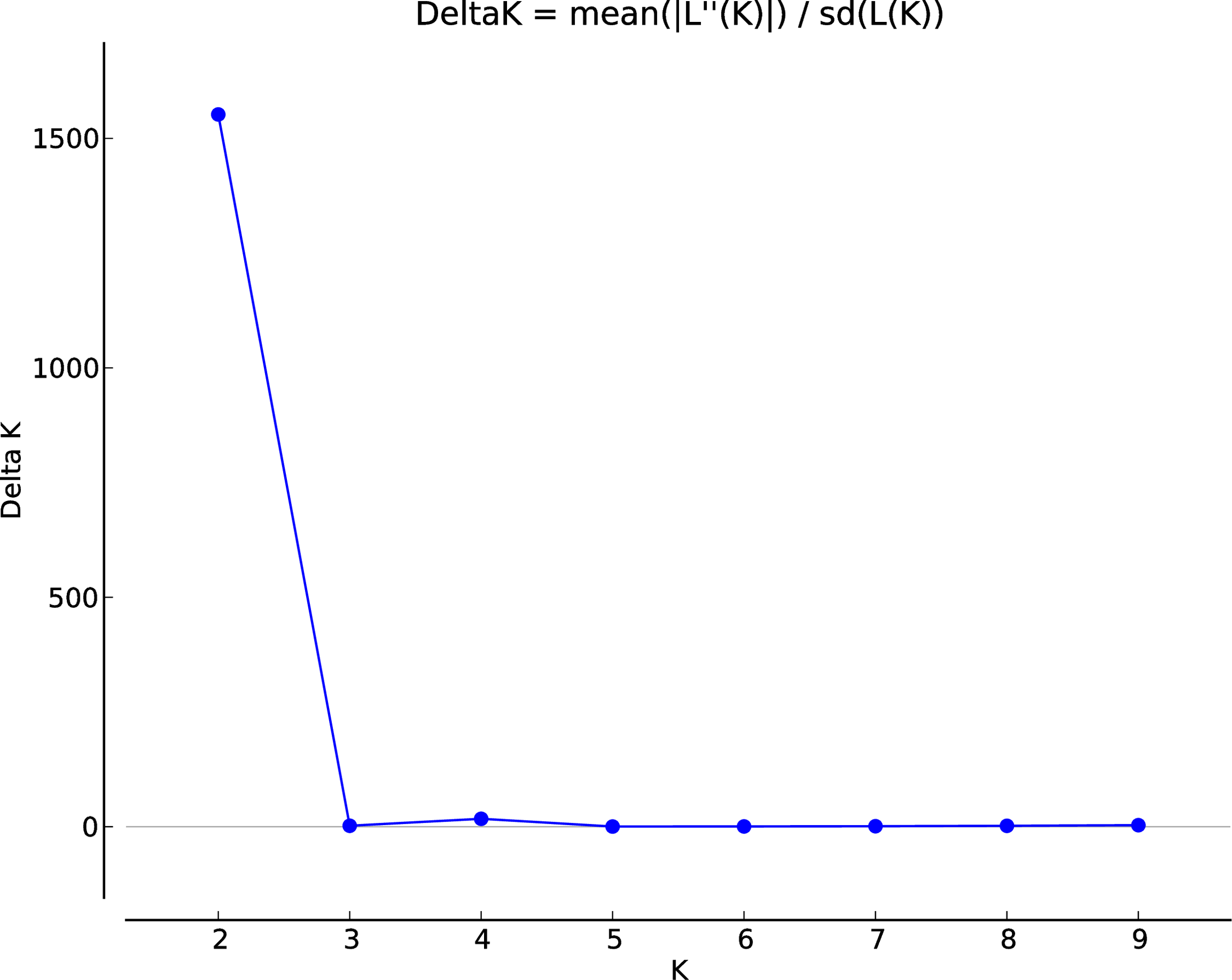

Supplement: S2 Fig — The Evanno method implemented in the STRUCTURE HARVESTER program suggested K = 2 as the most likely K value. (TIF) [file pone.0239123.s002.tif]

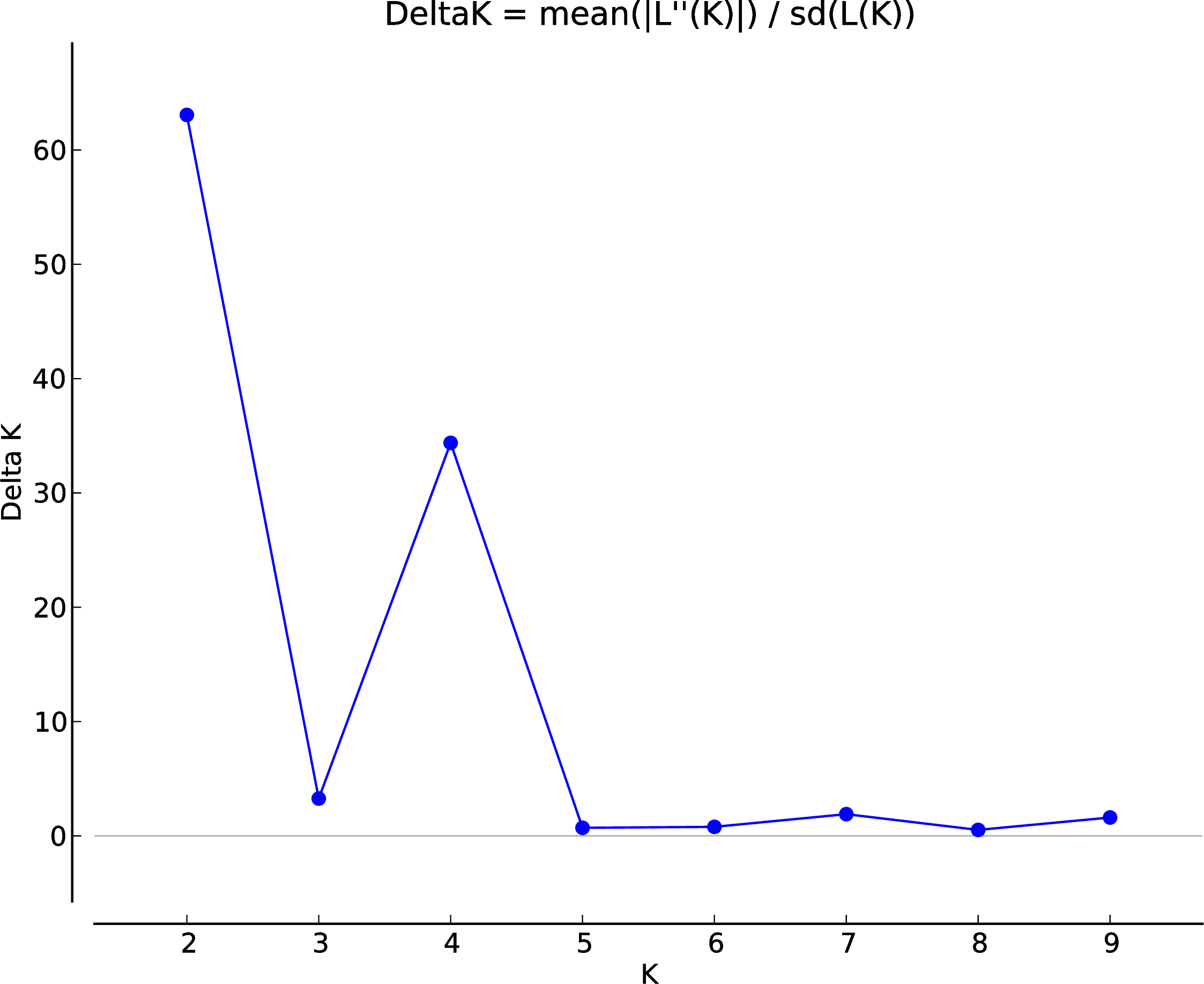

Supplement: S3 Fig — The Evanno method implemented in the STRUCTURE HARVESTER program suggested K = 2 as the most likely K value. (TIF) [file pone.0239123.s003.tif]

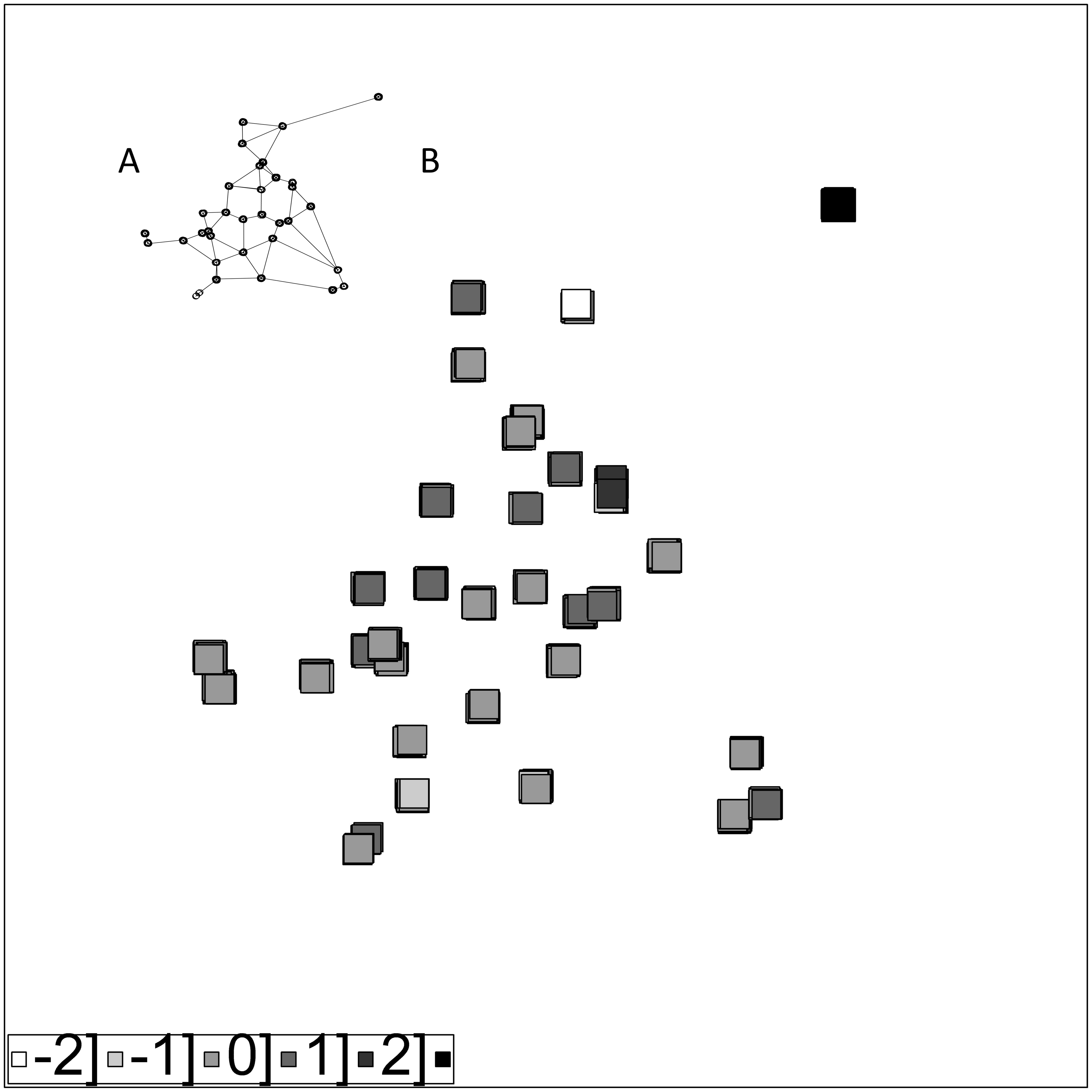

Supplement: S4 Fig — (A) Representation of the connexion network (Gabriel graph) used for the sPCA analysis. (B) Plot of the first sPCA axis scores. Scores are depicted over a grey level gradient, with dark grey and white colors for positive and negative scores, respectively. (TIF) [file pone.0239123.s004.tif]

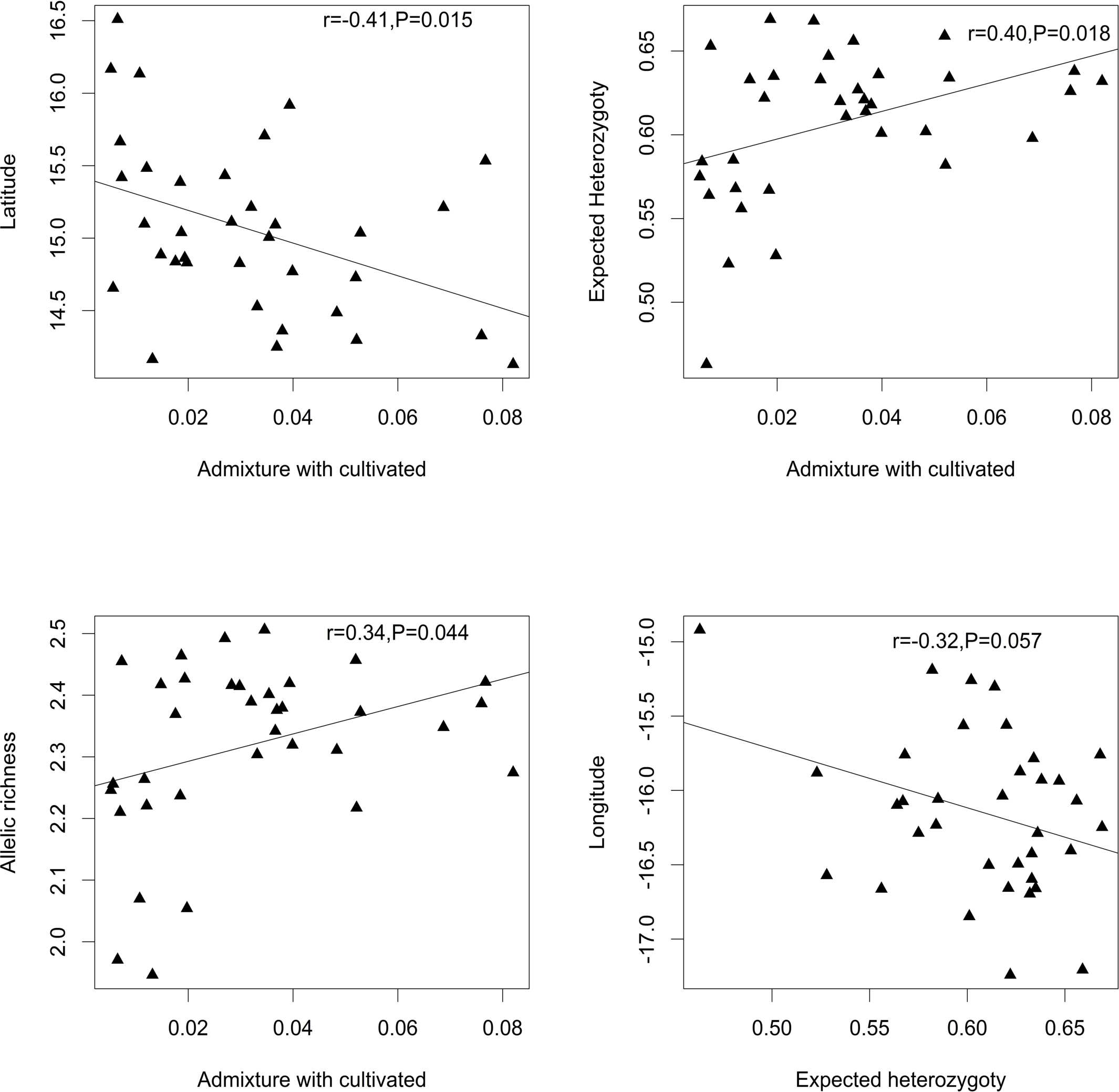

Supplement: S5 Fig — (TIF) [file pone.0239123.s005.tif]

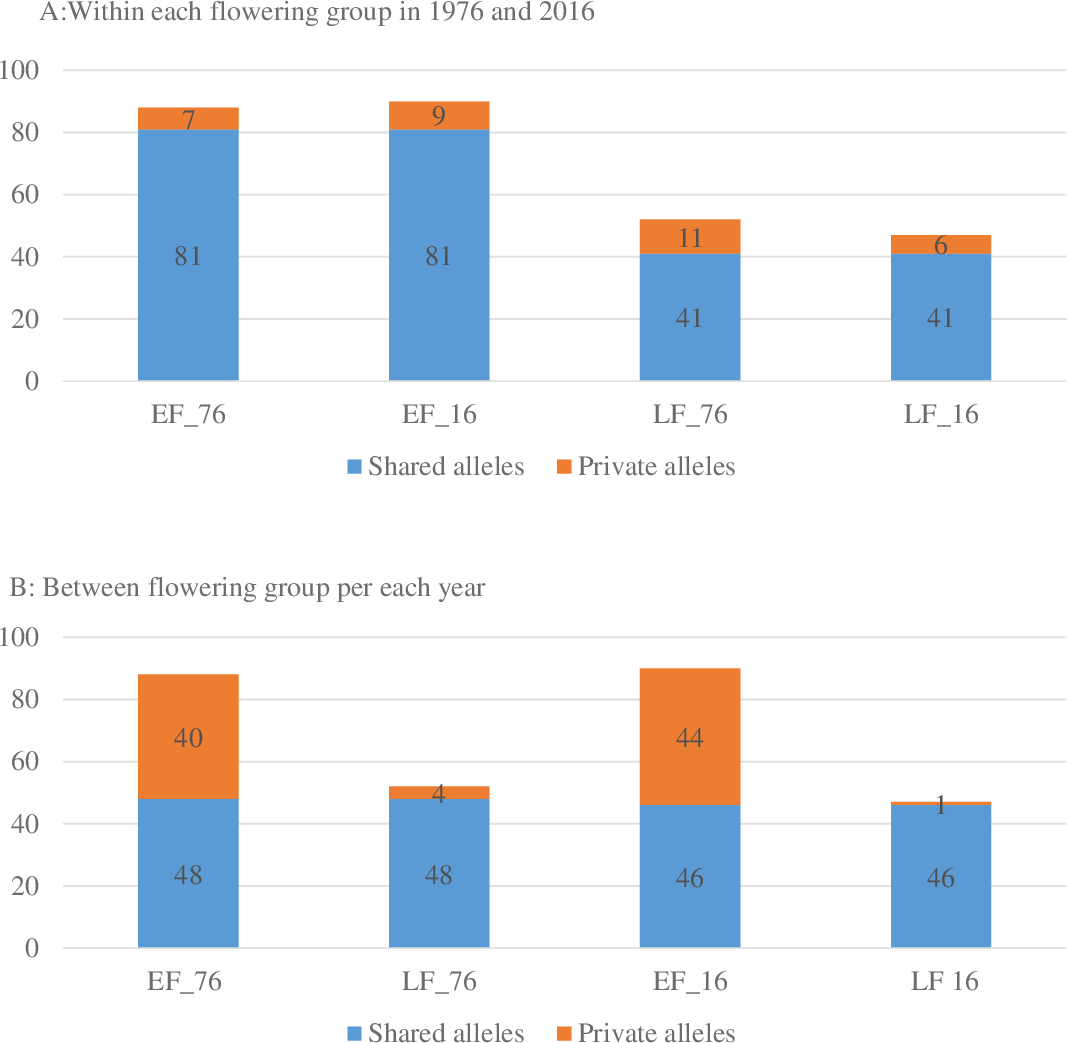

Supplement: S6 Fig — (TIF) [file pone.0239123.s006.tif]
