# Supplementary material for: Abandonment of pearl millet cropping and homogenization of its diversity over a 40 year period in Senegal
Source: PLoS One. 2020 Sep 14;15(9):e0239123. doi: 10.1371/journal.pone.0239123 (PMC7489563; doi:10.1371/journal.pone.0239123)
Supplement: S3 Table — (PDF) [file pone.0239123.s009.pdf]

S3 Table. Pearson correlation matrix between geographical coordinates and genetics estimates in wild and cultivated pearl millet according to sampling year.

		Latitude	Longitude	H_{Exp}	H_{Obs}	A_R	F_{IS}
**Wild**	**Latitude**						
	**Longitude**	0.26(0.136)					
	**H_{Exp}**	-0.31(0.072)	**-0.32(0.057)**				
	**H_{Obs}**	-0.26(0.125)	-0.25(0.140)	**0.78(0.000)**			
	**A_R**	-0.11(0.524)	-0.17(0.315)	**0.93(0.000)**	**0.78(0.000)**		
	**F_{IS}**	0.01(0.966)	0.01(0.955)	-0.11(0.537)	-0.17(0.341)	-0.14(0.434)	
	**q_{cw}**	**-0.41(0.015)**	0.04(0.840)	**0.4(0.018)**	0.3(0.083)	**0.34(0.044)**	-0.26(0.124)
**Cultivated in 1976**		**Latitude**	**Longitude**	**H_{Exp}**	**H_{Obs}**	**A_R**	**F_{IS}**
	**Latitude**						
	**Longitude**	0.53(0.064)					
	**H_{Exp}**	**0.77(0.002)**	0.34(0.261)				
	**H_{Obs}**	**0.68(0.011)**	0.46(0.118)	**0.70(0.007)**			
	**A_R**	**0.76(0.002)**	0.48(0.097)	**0.94(0.000)**	**0.71(0.007)**		
	**F_{IS}**	-0.01(0.979)	-0.31(0.308)	0.25(0.403)	-0.49(0.092)	0.16(0.590)	
	**q_{wc}**	-0.03(0.923)	0.02(0.940)	0.17(0.589)	0.10(0.746)	-0.14(0.650)	-0.26(0.398)
**Cultivated in 2016**		**Latitude**	**Longitude**	**H_{Exp}**	**H_{Obs}**	**A_R**	**F_{IS}**
	**Latitude**						
	**Longitude**	0.53(0.064)					
	**H_{Exp}**	0.45(0.125)	0.05(0.879)				
	**H_{Obs}**	**0.64(0.019)**	0.32(0.280)	**0.68(0.010)**			
	**A_R**	0.35(0.237)	0.32(0.289)	**0.84(0.004)**	0.67(0.012)		
	**F_{IS}**	-0.50(0.079)	-0.36(0.226)	-0.36(0.227)	-0.92(0.000)	-0.47(0.105)	
	**q_{wc}**	0.36(0.227)	-0.08(0.790)	0.50(0.079)	0.07(0.830)	0.278(0.352)	0.39(0.188)

H_{Exp}: expected heterozygosity; HObs: observed heterozygosity; AR: allelic richness; FIS: inbreeding coefficient, q_{wc}: mean admixture from cultivated to wild form, qcw: mean admixture from cultivated to wild form. P-values associated with each pearson coefficient are in bracket. Significant P-values ($P < 0.05$) are shown in bold.
